# Diagnostic challenges in appendiceal mucocele mimicking acute appendicitis: a three-case experience

**DOI:** 10.1097/RC9.0000000000000516

**Published:** 2026-05-14

**Authors:** Abdulrahman Mohammed Abdulrahman Abouh, ShamesEldeen Amara Amer, Yousif Ibrahim Mohamed, Fathia Salim Basher Mohammed, Awab Khalid Ahmed, Haitham Abdalla Ali Ismail

**Affiliations:** a Department of Surgery, Faculty of Medicine and Health Sciences, University of Kordofan, El Obeid, Sudan; b Department of General Surgery, El Obeid Teaching Hospital, El Obeid, Sudan; c Sheikan College, Medicine Program, El-Obeid, Sudan; d Department of Obstetrics & Gynaecology, Faculty of Medicine and Health Sciences, University of Kordofan, El Obeid, Sudan

**Keywords:** acute appendicitis, appendiceal mucocele, carcinoid tumor, case series, right iliac fossa mass, surgical management

## Abstract

**Introduction and importance:**

Appendiceal mucocele is a rare pathological condition characterized by the cystic enlargement of the appendix due to mucus accumulation. Clinically, it can mimic acute appendicitis or, less commonly, neoplastic lesions such as carcinoid tumors, creating diagnostic uncertainty. Accurate recognition during surgery and intact removal are essential to prevent rupture and subsequent pseudomyxoma peritonei.

**Presentation of cases:**

We describe three middle-aged female patients who presented with symptoms suggestive of acute appendicitis. The first case was a 40-year-old woman with right lower quadrant pain, nausea, and low-grade fever. The second case, a 36-year-old woman, presented with similar symptoms and was found intraoperatively to have a mass resembling a carcinoid tumor. The third patient, a 50-year-old diabetic woman, presented with right lower quadrant pain, vomiting, and fever. All underwent open appendectomy with intact removal of the appendix. Histopathology confirmed benign mucocele in all cases, with no evidence of malignancy. Postoperative recovery was uneventful, and all patients remained symptom-free during follow-up.

**Clinical discussion:**

These cases highlight the diagnostic challenges associated with appendiceal mucocele, which can mimic both inflammatory and neoplastic appendiceal conditions. Despite advances in imaging, preoperative diagnosis is often difficult, particularly in low-resource settings. Careful surgical technique to prevent rupture, combined with histopathological evaluation, is essential for accurate diagnosis and optimal prognosis.

**Conclusion:**

Although uncommon, appendiceal mucocele should be considered in patients with atypical appendicitis or a right iliac fossa mass. Recognizing its variable presentations and distinguishing it from carcinoid tumors are crucial to guide safe surgical management and prevent complications.

## Introduction

Appendiceal mucocele is an uncommon but clinically important entity, accounting for less than 0.3% of all appendectomy specimens^[^1,2^]^. It is characterized by progressive dilation of the appendix caused by mucus accumulation within the lumen. This condition can arise from benign processes, such as simple retention cysts or mucosal hyperplasia, as well as from neoplastic lesions, including low-grade appendiceal mucinous neoplasms and mucinous adenocarcinomas^[^1,3^]^. The diverse histopathology and the potential for peritoneal spread, particularly following rupture, make appendiceal mucocele a diagnostic and therapeutic challenge.

HIGHLIGHTS

- Appendiceal mucocele is an uncommon condition that can present similarly to acute appendicitis, complicating the diagnostic process.
- Preoperative imaging often cannot reliably differentiate a mucocele from other appendiceal or pelvic pathologies, highlighting the need for careful clinical assessment.
- Surgical excision with preservation of the appendix’s integrity is critical to prevent pseudomyxoma peritonei.
- Histopathological evaluation remains the definitive method to confirm a diagnosis and rule out malignant lesions.
- This case series from a resource-limited setting demonstrates safe surgical management and emphasizes clinicians’ awareness.
- One patient’s mucocele was initially mistaken for a carcinoid tumor during surgery, illustrating intraoperative diagnostic challenges.

Clinically, appendiceal mucocele often presents with nonspecific symptoms and may resemble acute appendicitis or manifest as a palpable right iliac fossa mass^[^4,5^]^. Preoperative diagnosis is uncommon, especially in settings where advanced imaging modalities, such as computed tomography (CT), are not readily available. While ultrasonography may identify a cystic, tubular, or ovoid structure in the right lower quadrant, its specificity is limited^[^1,4^]^. Consequently, many cases are discovered incidentally during surgery for presumed appendicitis.

Recent literature underscores the importance of recognizing mucocele intraoperatively and removing it intact to prevent rupture and subsequent pseudomyxoma peritonei, a potentially life-threatening complication^[^3,5,6^]^. The choice of surgical approach remains debated: although laparoscopic appendectomy has been increasingly reported, open surgery is often preferred when the diagnosis is uncertain to minimize the risk of spillage^[^5,6^]^.

We report three cases of appendiceal mucocele managed in which all patients presented with clinical features suggestive of acute appendicitis. In one case, intraoperative findings raised suspicion of a carcinoid tumor, but histopathology confirmed a benign mucocele (Table 1). This series highlights the diagnostic challenges in low-resource settings and emphasizes the importance of careful surgical technique and histopathological confirmation to ensure accurate diagnosis and safe management. This case series has been reported in line with the PROCESS 2025 criteria^[^7^]^.

**Table 1:** Baseline clinical characteristics of the three patients included in this case series of appendiceal mucocele.

Characteristic	Case 1	Case 2	Case 3
Age (years)	40	36	50
Sex	Female	Female	Female
Comorbidities	None	None	Diabetes mellitus, hypothyroidism
ASA score	ASA II	ASA II	ASA II
Presenting symptoms	Right lower quadrant pain, nausea, mild fever	Right lower quadrant pain, fever, nausea	Right lower quadrant pain, fever, nausea, vomiting
Duration of symptoms	2 days	1 day	4 days
Ultrasound findings	Dilated appendix	Appendicular mass 3 × 4 cm	Features of acute appendicitis
Type of surgery	Emergency open appendectomy	Emergency laparotomy	Emergency open appendectomy
Histopathology	Mucocele (benign)	Mucocele (benign)	Mucocele (benign)

## Case presentations

A combined clinical timeline of the three cases is presented in Table 2, illustrating the sequence of events from symptom onset through diagnosis, surgical intervention, and postoperative recovery.

**Table 2:** Sequence of events from symptom onset through diagnosis, surgical intervention, and postoperative recovery.

Day	Case 1	Case 2	Case 3
Day 1	Onset of periumbilical abdominal pain	Central abdominal pain → migrated to right lower quadrant, anorexia, fever, nausea	Epigastric pain → migrated to right lower quadrant
Day 2	Pain migrated to right lower quadrant, anorexia, low-grade fever, nausea		
Day 3	Clinical exam, labs, ultrasound → emergency open appendectomy; mucocele identified and sent for histopathology	Clinical exam, labs, ultrasound → emergency laparotomy; appendicular mass (3 × 3 cm, suspected carcinoid tumor)	
Day 4	Postoperative recovery	Postoperative recovery	Fever and vomiting
Day 5	Continued recovery → discharged	Postoperative recovery	Clinical exam, labs, ultrasound → emergency open appendectomy; mucocele identified and sent for histopathology
Day 6		Discharged	Postoperative recovery
Day 7			Discharged

### Case 1

A 40-year-old female presented with periumbilical pain that migrated to the right iliac fossa over 2 days, associated with loss of appetite, nausea, and mild fever. She had four children, a regular menstrual cycle, and her last menstrual period was 10 days prior. There were no urinary or gastrointestinal symptoms.

Examination: She appeared mildly unwell and febrile, without pallor, jaundice, or cyanosis. Chest and cardiovascular examinations were normal. Abdominal examination revealed tenderness and rebound tenderness in the right lower quadrant, without palpable masses.

Investigations:

- Hemoglobin: normal
- Total white blood cells (WBC): 10 000/mm^3^ (neutrophilia)
- C-reactive protein: normal
- Urine analysis: clear
- Serum human chorionic gonadotropin (hCG): negative
- Renal function tests: normal
- Abdominal ultrasound: dilated appendix with a diameter of 1 cm, no collection or abscess.

**Figure 1.**
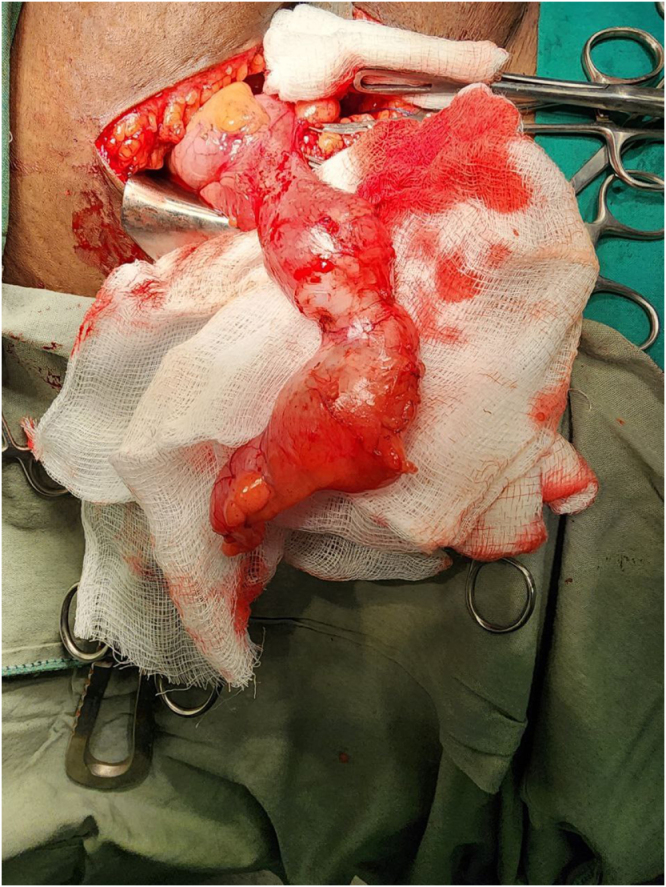
Intraoperative picture showing dilated appendix from Case 1.

**Figure 2.**
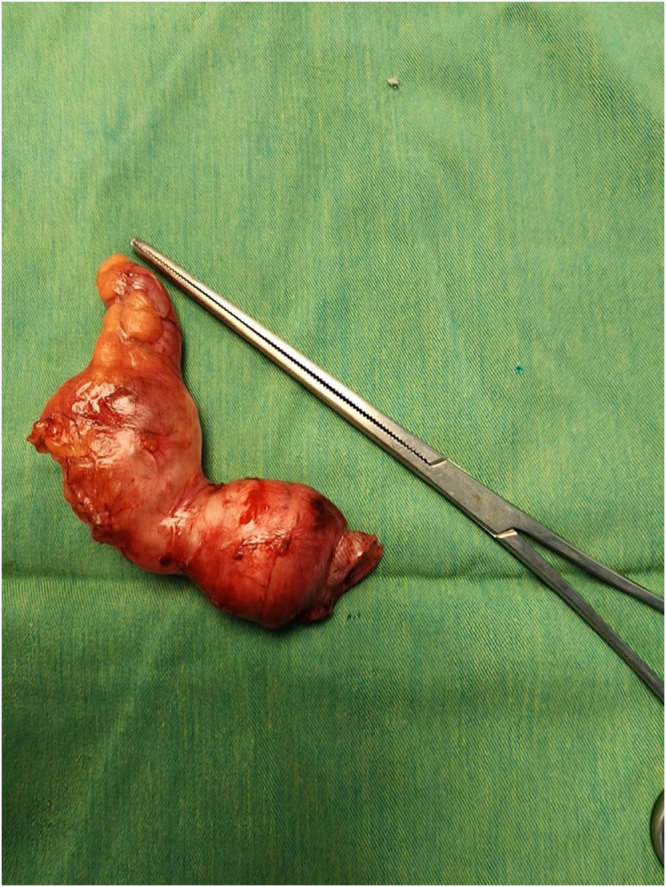
Gross specimen from Case 1 showing fusiform cystic enlargement of the appendix, consistent with an appendiceal mucocele.

Management and operative findings:

The patient was diagnosed with acute appendicitis and underwent an open appendectomy via a Gridiron incision. Intraoperative findings revealed a dilated appendix filled with mucus, consistent with a mucocele (Fig. 1). The appendix was removed intact and sent for histopathology (Fig. 2).

Histopathology: Mucocele of the appendix (mucinous cystic dilatation without malignancy).

Outcome: Postoperative recovery was uneventful. The patient was discharged on the 2nd postoperative day with antibiotics and followed up regularly every 2 weeks for 6 weeks, remaining asymptomatic.

### Case 2

A 36-year-old female presented with central abdominal pain that shifted to the right iliac fossa over one day. The pain was aggravated by movement and relieved by lying still. She experienced nausea but no vomiting, abdominal distension, or constipation. She had no history of diabetes, hypertension, asthma, or prior abdominal surgery, and was not on any regular medication. Obstetric history revealed gravida five, para zero, with regular menstrual cycles and no contraceptive use.

On examination, the patient appeared unwell and febrile but was not pale, jaundiced, or cyanosed. Her pulse and blood pressure were within normal limits, and the cardiopulmonary examination was unremarkable. Abdominal examination revealed a normal contour with diminished movement on respiration. There was localized tenderness and rebound tenderness in the right iliac fossa, without organomegaly or a palpable mass.

Laboratory investigations showed leukocytosis with otherwise normal hematologic indices. Urinalysis revealed a few pus cells (4–10 per high-power field) with no significant bacteriuria. Abdominal ultrasonography demonstrated a 3 × 4 cm appendicular mass, with other intra-abdominal organs appearing normal.

**Figure 3.**
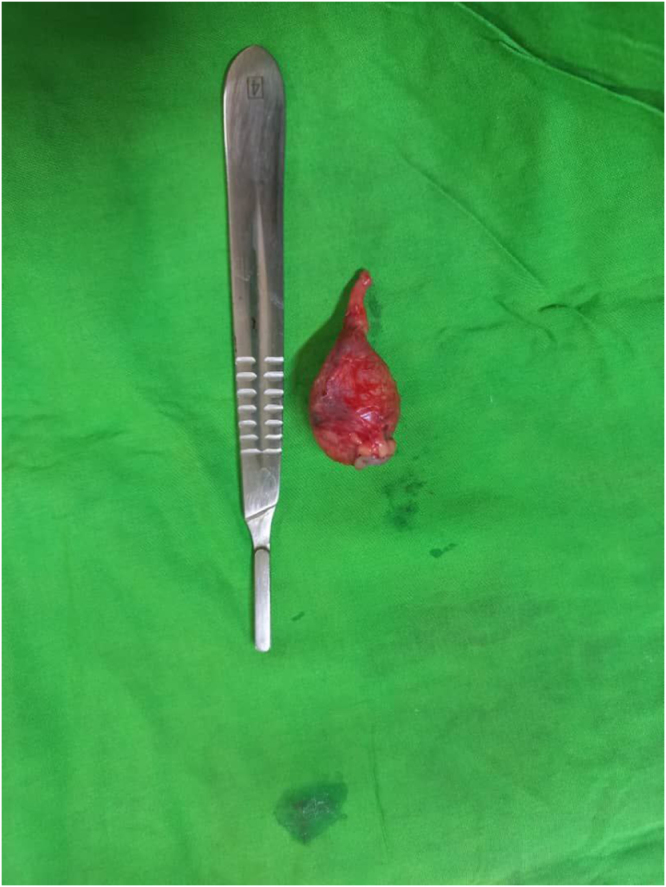
Resected appendix from case 2 with prominent distal dilatation and intact serosal surface, consistent with appendiceal mucocele.

The patient was prepared for surgery and underwent exploratory laparotomy via a lower midline incision. Intraoperatively, a rounded, cystic mass measuring approximately 3 × 3 cm was identified in the distal half of the appendix. There were no enlarged lymph nodes or evidence of peritoneal spread. Appendectomy was performed, and the specimen was sent for histopathological evaluation. The intraoperative impression was a carcinoid tumor of the appendix (Fig. 3).

The postoperative course was uneventful, and the patient recovered well. Histopathological examination, however, revealed a mucocele of the appendix without malignant cells, confirming a benign lesion.

### Case 3

A 50-year-old female with diabetes and hypothyroidism presented with epigastric pain that migrated to the right lower quadrant over 4 days, accompanied by fever and vomiting.

Investigations:

- Hemoglobin: 12 grams
- Total WBC: 11 000/mm^3^ (neutrophilia)
- Urine analysis: pus cells 4–6
- Renal function tests: normal
- Random blood glucose: 160 mg/dL
- Abdominal ultrasound: suggested acute appendicitis
- Normal chest X-ray and electrocardiogram.

**Figure 4.**
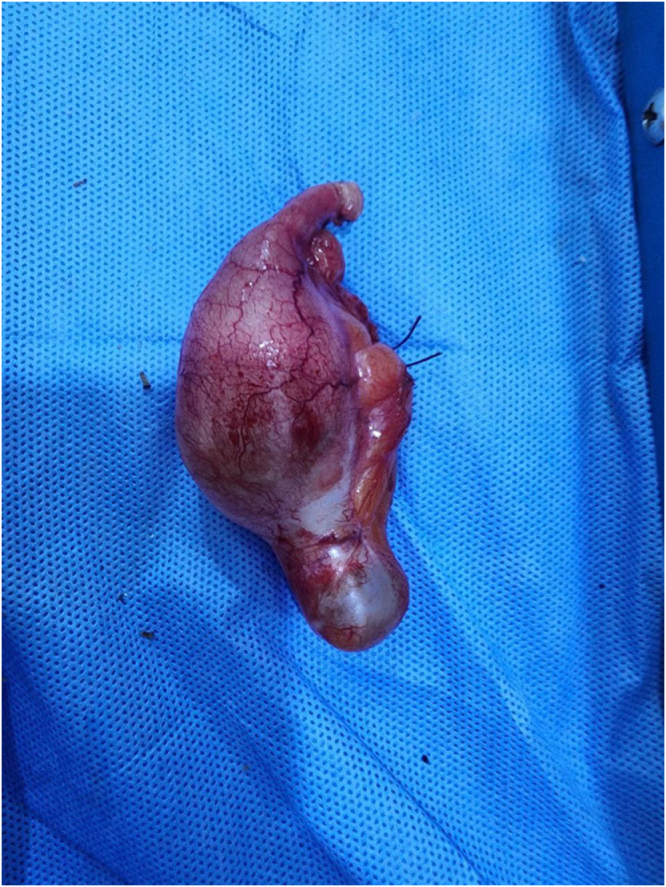
Specimen from Case 3 showing bulbous cystic enlargement of the appendix, compatible with a benign appendiceal mucocele.

Management and operative findings:

The patient was diagnosed with acute appendicitis and underwent an open appendectomy via a Gridiron incision. Intraoperative findings revealed a 10-cm dilated appendix. The appendix was removed intact and sent for histopathology (Fig. 4).

Histopathology: mucocele of the appendix (mucinous cystic dilatation without malignancy).

Outcome: Postoperative recovery was uneventful. The patient was discharged on the 2nd postoperative day with antibiotics and followed up regularly every 2 weeks for 6 weeks, remaining asymptomatic.

## Method

This case series has been reported in line with the PROCESS 2025 criteria. A completed PROCESS 2025 criteria is submitted as a Supplemental Digital Content file, available at: http://links.lww.com/IJSCR/A51 alongside this manuscript.

## Discussion

With modern imaging and minimally invasive surgery, mucoceles of the appendix continue to evolve, ranging from simple retention cysts to cystadenocarcinoma. Although this condition was first described over a century ago, diagnostic and therapeutic challenges remain the same. In recent literature, difficulties persist in preoperative identification, and many cases are discovered incidentally during imaging or surgery for suspected appendicitis^[^1,4,5,8^]^.

Clinically, it is often nonspecific, with the right lower quadrant pain mimicking acute appendicitis or adnexal pathology in females^[^2,6^]^. As an inadvertent rupture can lead to pseudomyxoma peritonei (PMP), an extremely serious complication with significant morbidity, a meticulous intraoperative assessment is essential^[^1^]^. Despite appearing as benign lesions, oncologically sound approaches are warranted even when there is a risk of malignant transformation or coexisting neoplasia^[^3^]^.

Diagnostic imaging modalities, such as ultrasound and CT, play a crucial role in diagnosis. MRI may provide specificity in equivocal cases, especially when distinguishing mucocele from adnexal cysts or duplication cysts^[^8,9^]^, although the onion-skin sign on ultrasound and low-attenuation cystic dilation on CT are not pathological^[^4,10^]^. It is, however, not sufficient to use imaging alone to determine the risk of malignancy, so surgical excision and histopathological confirmation are necessary ^[^4^]^.

Surgical management has transitioned toward individualized, minimally invasive strategies. While open surgery remains the standard in suspected malignant or giant mucoceles, laparoscopic resection has proven safe in well-selected benign cases when performed with gentle handling and avoidance of rupture^[^1,5,9^]^. To prevent dissemination, intact specimen retrieval and peritoneal washout are essential^[^6^]^. Currently, there is a debate about right hemicolectomy versus appendectomy alone; most recent studies advocate a customized approach based on intraoperative findings and histopathology^[^10,11^]^.

Coexistence with neuroendocrine tumors of the appendix underscores the need for a thorough histological evaluation^[^12^]^. Rare associations have been documented, broadening the clinical spectrum of appendiceal mucocele. The diagnosis of appendiceal intussusception, which mimics a mucocele, can be challenging with advanced imaging techniques^[^13^]^. These rare variants emphasize the necessity of maintaining a high index of suspicion and considering mucocele in the differential diagnosis of right lower quadrant masses.

Our three cases further reinforce these evolving paradigms. They were discovered incidentally during surgery for presumed appendicitis, reflecting the diagnostic limitations in resource-limited settings. It was safe and feasible to perform laparoscopic resection, supporting the growing body of evidence that minimally invasive surgery, when performed cautiously, can result in excellent results^[^1,6,9^]^. However, intraoperative decision-making, specimen integrity, and histopathological guidance remain crucial to successful management.

Because of its anatomical proximity to the right adnexa, an appendiceal mucocele can easily mimic a right ovarian cyst or mass on ultrasound or CT/MRI. Several reports in the gynecologic surgery literature have described cases where appendiceal mucoceles were initially misdiagnosed as ovarian cysts during preoperative evaluation. Moreover, there is a well-documented association between appendiceal mucinous neoplasms and ovarian mucinous tumors^[^14,15^]^.

## Conclusion

Appendiceal mucocele, though uncommon, should be considered in patients with atypical or mass-forming appendicitis. Recognition of its varied presentation and differentiation from carcinoid tumor are essential to ensure optimal surgical management and prevent complications.

## Data Availability

The data that support the findings of this study are available from the corresponding author upon reasonable request.

## Research registration unique identifying number (UIN)

Name of the registry: Research Registry. Unique identifying number or registration ID: researchregistry11641. Hyperlink to your specific registration: https://www.researchregistry.com/browse-theregistry#home.

## Patient perspective

While advanced imaging tools were not available for the diagnostic process, the patients expressed gratitude for the prompt diagnosis and surgical treatment they received. They were relieved to learn the reason for their symptoms and satisfied with the care they received throughout the hospitalization and recovery process.
